# Efficient genome editing in *Caenorhabditis elegans* by CRISPR-targeted homologous recombination

**DOI:** 10.1093/nar/gkt805

**Published:** 2013-09-05

**Authors:** Changchun Chen, Lorenz A. Fenk, Mario de Bono

**Affiliations:** Division of Cell Biology, MRC Laboratory of Molecular Biology, Francis Crick Avenue, Cambridge CB2 0QH, UK

## Abstract

Cas9 is an RNA-guided double-stranded DNA nuclease that participates in clustered regularly interspaced short palindromic repeats (CRISPR)-mediated adaptive immunity in prokaryotes. CRISPR–Cas9 has recently been used to generate insertion and deletion mutations in *Caenorhabditis elegans,* but not to create tailored changes (knock-ins). We show that the CRISPR–CRISPR-associated (Cas) system can be adapted for efficient and precise editing of the *C. elegans* genome. The targeted double-strand breaks generated by CRISPR are substrates for transgene-instructed gene conversion. This allows customized changes in the *C. elegans* genome by homologous recombination: sequences contained in the repair template (the transgene) are copied by gene conversion into the genome. The possibility to edit the *C. elegans* genome at selected locations will facilitate the systematic study of gene function in this widely used model organism.

## INTRODUCTION

Clustered regularly interspaced short palindromic repeats (CRISPR) and CRISPR-associated (Cas) proteins provide eubacteria and archaea with an adaptive defense system against invading viral and plasmid DNA (1,2). CRISPR RNAs (crRNAs), in complex with *trans*-activating crRNA (tracrRNA) and Cas proteins, guide sequence-specific cleavage of foreign nucleic acids, preventing their proliferation and propagation (3). Cleavage following Cas9-mediated DNA unwinding requires both complementarity between the crRNA and a target sequence, and the presence of a short motif termed protospacer adjacent motif (PAM) (4). Recent *in vitro* work using the *Streptococcus pyogenes* type II CRISPR–Cas system has shown that a synthetic single guide RNA (sgRNA), consisting of a fusion of crRNA and tracrRNA, is able to direct the Cas9 endonuclease to introduce targeted double-strand breaks (4). This two-component system has since been adapted to induce targeted double-strand breaks in several heterologous systems, including cultured human cells (5,6), mice (7,8), zebrafish (9), *Drosophila* (8), bacteria (10) and, most recently, *Caenorhabditis elegans* (11). The CRISPR–Cas9 system’s potential to target a genomic interval appears to be limited only by the requirement for an NGG PAM sequence, making it highly versatile as a genome-editing tool.

The ability to generate targeted knock-outs and knock-ins has provided powerful ways to study gene function in model organisms such as yeast (12), mice (13,14) and flies (15–18). Knock-in methods are particularly versatile, as they enable proteins to be modified at specific residues, and tagged by addition of fluorescent proteins or antibody epitopes. These approaches rely on homologous recombination between engineered DNA and the targeted locus. In *C. elegans*, the rate of homologous recombination is inefficient (19,20–23), and must be stimulated to be of routine use. The state-of-the-art method to do this involves hopping out a *Mos*1 transposon from the locus to be engineered by expressing *Mos*1 transposase in the germline (24). This creates a double-strand break that is frequently repaired by gene conversion using engineered transgenes present in *trans* as templates. A limitation of this approach, which is called *Mos1* excision-induced transgene-instructed gene conversion (MosTIC), is that it requires a strain bearing a *Mos*1 transposon inserted in the appropriate location. To meet this requirement, large strain collections have been accumulated harboring *Mos*1 at different genomic intervals (25). Despite these efforts, a significant fraction of genes (∼60%) are currently too far from a *Mos*1 insertion site to be efficiently edited (25). This is a particular problem, as the efficiency of gene conversion declines steeply with distance from the double-strand break (24). The ability to target CRISPR–Cas9-induced double-strand breaks anywhere in the genome, limited only by the availability of the PAM sequence NGG, potentially offers a way round this limitation.

Here, we show that double-strand breaks can be engineered at precise locations in the *C. elegans* genome by injecting the core components of the prokaryotic type II CRISPR–Cas adaptive immune system. Non-homologous end-joining of these breaks efficiently generates small deletions and insertions in the region, resulting in gene knockout. The double-strand breaks can be used for transgene-instructed gene conversion. This allows customized changes in the *C. elegans* genome by homologous recombination: sequence variations contained in the repair template (the transgene) are copied by gene conversion into the genome. The possibility to edit the *C. elegans* genome efficiently and, at least in principle, anywhere will facilitate the systematic study of gene function in this widely used model organism.

## MATERIALS AND METHODS

### Strains

Strains used include N2 and *ben-1(e1880) III*. Animals were maintained as described previously (26).

### Plasmids and molecular biology

**Figure 1.**
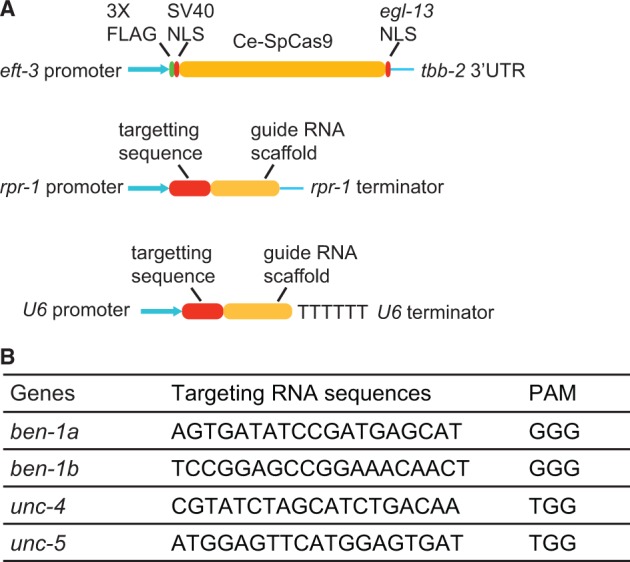
CRISPR–Cas targeting in *C. elegans.* (**A**) Vectors optimized to express Cas9 and sgRNA in the *C. elegans* germline. NLS, nuclear localization signal. (**B**) Targeting sequences used for different genes, together with the 3′ PAM sequence.

The Cas9 open reading frame was codon-optimized for *C. elegans* (27), and modified by inserting a 3X FLAG tag and an SV40 nuclear localization sequence (NLS) after the ATG start codon, and a further NLS from the *egl-13* gene just upstream of the stop codon (Figure 1A). This artificial gene, called *Ce Cas9*, was synthesized from oligonucleotides (GeneArt, Life Technologies). To drive expression of *Ce Cas9* in *C. elegans*, we used the *eft-3* promoter. We also placed the *tbb-2* 3′UTR after the Cas9 open reading frame (Figure 1A). The *eft-3* promoter and *tbb-2* 3′UTR have been used previously to optimize germline expression of transposase in MosTIC protocols (24). The *peft-3::Cas9::tbb-2* 3′UTR construct was assembled using the multisite Gateway system (Life technologies). This plasmid will be made available via Addgene (http://www.addgene.org).

We expressed chimeric single guide RNAs (6) under the control of either the *rpr-1* or U6 promoters. We inserted an *Eco* RI site between the *rpr-1* promoter and the guide RNA scaffold, to facilitate cloning of targeting RNA sequences using the Gibson assembly kit (New England Biolabs). Briefly, two complementary oligos, in which the targeting RNA sequence was flanked by the 15-bp sequences just outside *Eco* RI site, were annealed and mixed with *Eco* RI-digested plasmid and the Gibson assembly master mix. The reaction mix was incubated at 50°C for 30 min and transformed into competent cells. The resulting plasmids were sequenced to confirm insertion of the targeting RNA sequence. Targeting oligos can be designed as follows: GCGCGTCAAGTTGTG NNNNNNNNNNNNNNNNNNN GTTTTAGAGCTAGAA, where N represents a 19-base targeting sequence from the genomic region of interest that is adjacent to a PAM sequence. The plasmid with the U6 promoter contains a unique *Hin* dIII site between U6 promoter and the guide RNA scaffold. To insert the targeting RNA sequence into this vector, the following oligo format can be used: ATTTCATACAAATTG NNNNNNNNNNNNNNNNNNN GTTTTAGAGCTAGAA.

Expression of the hygromycin-resistance gene (HygR; a gift from Jason Chin) (28) was driven by the *rps-0* promoter, and a *prps-0::HygR::unc-54* 3′UTR cassette was inserted in the second position of a pENTRY vector of the multisite Gateway system. Flanking regions containing ∼2 kb of homologous DNA from either side of the targeted *ben-1* locus were put into the first and third position, respectively.

To verify mutations obtained using CRISPR, DNA was extracted from mutant F2 animals, and the targeted gene amplified by polymerase chain reaction (PCR) and sequenced.

### Transgenic animals

N2 animals were grown using standard conditions before micro-injection (29). The plasmid carrying *Ce Cas9* was injected at either 30 ng/μl or 3 ng/μl together with 100 ng/μl of guide RNA construct and 30 ng/μl of coelomocyte green fluorescent protein (GFP) marker (cc::GFP). The higher concentration of *Ce Cas9* plasmid consistently gave better results.

### Benomyl assay

The assay was performed as described (30,31). Injected animals were transferred to plates containing 7 μM benomyl and maintained at 25°C. F1 animals expressing the co-injection marker (cc::GFP) were picked onto fresh benomyl-containing plates and their F2 progeny was scored for benomyl resistance by touching them at the anterior to provoke movement. Non-paralyzed worms were counted as resistant.

### Integration of the HygR gene

To insert the HygR gene into the *ben-1* locus, we adapted a protocol used in MosTIC transgenesis (32). We co-injected 30 ng/μl of the plasmid carrying *Ce Cas9*, 100 ng/μl of the *ben-1a* or *ben-1b* guide RNA construct, 30 ng/μl of the plasmid containing the HygR gene and homologous flanking sites, 10 ng/μl *phsp-16.41::peel-1*, 10 ng/μl *prab-3::mcherry*, 2.5 ng/μl *pmyo-2::mcherry* and 5 ng/μl *pmyo-3::mcherry*. For the insertion at *ben-1a* site, injected N2 animals were placed on plates containing 7 μM benomyl and grown at 25°C. Once the F1 progeny had yielded many F2 larvae, we added hygromycin to the plates to a final concentration of 0.2–0.3 mg/ml. Animals were heat-shocked for 2 h at 34°C after 2 days of hygromycin selection. The surviving animals were transferred to fresh hygromycin plates. To insert hygR gene into *ben-1b* site, injected N2 animals were placed on NGM plates until many F2 larvae were observed. Hygromycin was then added to the plate to achieve a final concentration of 0.2–0.3 mg/ml.

To confirm that targeted gene conversion had occurred, primers hybridizing to sequences located outside of the homologous flanking regions were used to amplify the modified gene. The resulting PCR products were sequenced.

## RESULTS

### CRISPR–Cas-directed mutagenesis

**Figure 2.**
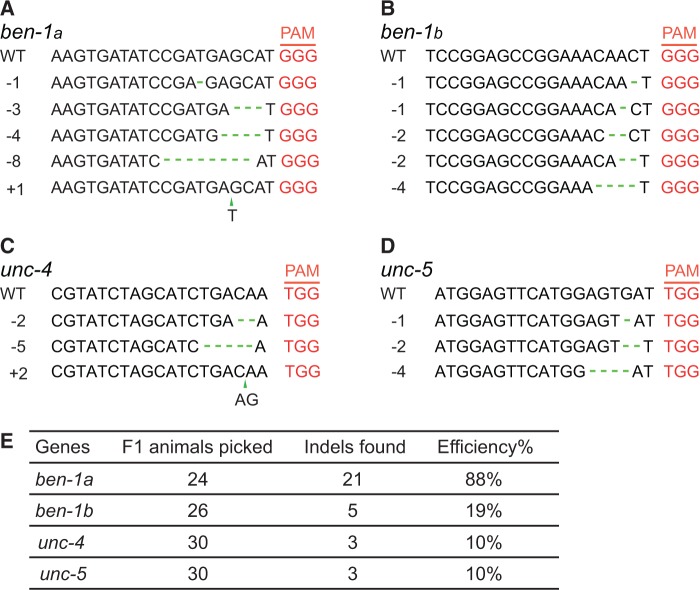
Efficiency of CRISPR–Cas-induced mutations. (**A–D**) Mutations induced by CRISPR–Cas9 using targeting sequences for *ben-1* (A and B), *unc-4* (C) and *unc-5* (D). For *ben-1a*, only a subset of the mutations we obtained is shown. (**E**) Efficiency of CRISPR-induced mutagenesis at different loci.

We sought to establish the use of CRISPR–Cas-targeted gene conversion in *C. elegans*. To assess the efficiency with which we could induce double-strand breaks at specific genomic locations, we first targeted the *ben-1* (*ben*omyl resistance-1) gene (30). Loss-of-function mutations in *ben-1* confer dominant resistance to the paralysis-inducing drug benomyl (30). We constructed an expression vector in which Cas9 endonuclease, codon-optimized for *C. elegans*, was driven from the *eft-3* promoter (Figure 1A). This promoter has previously been used effectively to drive germline transposase expression in *Mos1*-mediated transgenesis (32). To target Cas9 to the *ben-1* locus, we made a synthetic gene that expressed an sgRNA containing 20 bases of *ben-1* sequences under the control of either the *rpr-1* or *U6* promoters (Figure 1A). We injected the *Ce* Cas9 and sgRNA constructs together with the *unc-122::GFP* co-injection marker (informally known as cc::GFP because it is expressed in coelomocytes) into the gonad of young adult worms. We selected transgenic progeny of the injected animals using the cc::GFP marker, placed them individually on plates containing 7 μM benomyl and scored their offspring for benomyl resistance. Twenty-one of 24 transgenic F1 animals (88%) expressing the guide RNA under the *rpr-1* promoter produced benomyl-resistant offspring. We sequenced the *ben-1* gene in 11 of the 21 benomyl-resistant lines and looked for mutations. We identified 11 independent indels comprising four insertions and seven deletions (Figure 2A). We obtained similar results when we drove expression of the guide RNA from the U6-promoter. In this case, 89% of transgenic F1 animals gave rise to resistant progeny (24 of 27). We also noticed that a substantial fraction of non–GFP-expressing F1 animals showed heritable benomyl resistance, and carried mutations at the predicted sites (data not shown). These results suggest efficient gene targeting by this *Ce* Cas/sgRNA combination.

Having successfully introduced mutations in *ben-1*, we explored the generalizability of CRISPR–Cas9 system by targeting additional genes. We focused on *unc-4* and *unc-5,* which are both required for proper motor neuron development and locomotion (33,34). For each gene, we found that ∼10% of GFP-expressing F1 animals produced uncoordinated progeny. Sequence analysis confirmed that we had introduced mutations in the designated regions (Figure 2C and D). All mutations we have introduced to date using the CRISPR–Cas system were short insertions or deletions within a few base pairs of the PAM site. Such mutations are typical of double-strand break repair by non-homologous end-joining. These data suggest that the CRISPR system can target double-strand breaks in all the *C. elegans* genes we tested.

### Different sgRNAs can vary in their ability to target the same gene

To investigate whether different guide sequences from the same gene varied significantly in their targeting efficiency, we expressed an sgRNA that targeted a different site in the *ben-1* gene. Of 26 transgenic F1 animals we picked, five yielded mutant lines that exhibited benomyl resistance, an efficiency of 19% (Figure 2B). Thus, this second sgRNA also worked well, but less efficiently than the first one (Figure 2D). Our data suggest that designing different sgRNAs can be useful to optimize targeting of specific genes.

### CRISPR–Cas-targeted homologous recombination using transgene templates

**Figure 3.**
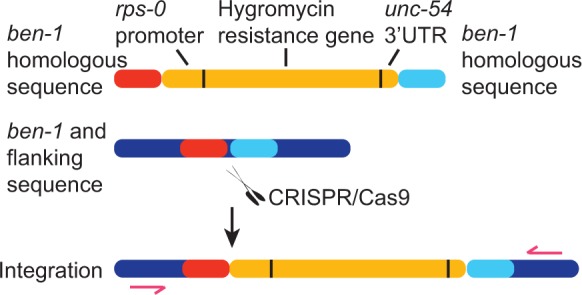
CRISPR-targeted knock-ins. Schematic showing targeted knock-in of an HygR cassette at the *ben-1* locus. The double-strand break induced at *ben-1* by CRISPR–Cas9 is repaired using the transgene containing the HygR cassette, leading to gene conversion.

Having demonstrated that the CRISPR–Cas system can efficiently introduce indels at specified regions of the *C. elegans* genome, we asked whether the double-strand breaks could be used to stimulate gene conversion of engineered transgenes by homologous recombination. Previous work has shown that the double-strand breaks created by excision of a *Mos*1 transposon can significantly stimulate transgene-mediated gene conversion in the vicinity of the *Mos*1 insertion site (24). We speculated that CRISPR–Cas-targeted double-strand break would do the same. Inspired by the yeast field, which uses drug selection to identify homologous recombination events, we sought to target an HygR gene to the *ben-1* locus, using the *ben-1*a sgRNA (Figures 1B and 3). The resistance to hygromycin B conferred by the HygR gene offers very efficient selection in C. elegans (35). We created a vector in which an HygR cassette was flanked by ∼2 kb of DNA sequence homologous to either side of the *ben-1a* site. We co-injected this vector with the *Ce* Cas9 and *ben-1*a sgRNA constructs (Figure 1A). We also added to the injection mix DNA encoding the PEEL-1 toxin (22) under the control of a heat-shock promoter (*phsp-16.41::peel-1*). This provided a way to select against animals bearing extrachromosomal arrays of the injected transgenes, facilitating identification of transgenic animals containing homologous integration of the hygromycin gene. Following gene conversion, such animals would have the HygR cassette but not the *phsp-16.41::peel-1* transgene. Negative selection using *peel-1* has been used efficiently before in MosTIC protocols (32). We injected 30 N2 animals and singled them to individual plates. When there were plenty of F2 progeny on the plates, we added hygromycin solution to the plates to select for HygR animals. From 30 injected P_o_ animals, we obtained three lines that were resistant to both hygromycin and benomyl. We expected resistance to benomyl, as insertion of the HygR cassette should disrupt the *ben-1* locus. As controls, we injected 50 animals with only the sgRNA-encoding plasmid, and another 50 animals with only the Cas9 plasmid. We were not able to detect any gene conversion events among their F2 progeny (as detected by HygR) in either experiment, suggesting efficient gene conversion required the CRISPR–Cas system.

To confirm that gene conversion had occurred, we PCR-amplified the *ben-1* region using primer pairs that flank sequences present in the transgene construct mediating repair (Figure 3). A PCR fragment of ∼9.4 kb, equivalent to the size of a single-copy hygromycin insertion, was obtained in all three lines (data not shown). We sequenced this entire fragment and found it was identical to the sequence provided on the repair transgene, confirming precise gene conversion.

Encouraged by the successful insertion of HygR gene into the *ben-1a* site, we reasoned that this method could facilitate the isolation of CRISPR–Cas9-induced gene conversion events that cause no obvious phenotypes. To demonstrate this, we used the existing reagents to target the HygR gene to the *ben-1b* site without benomyl selection. One HygR line was obtained out of 53 injected N2 animals. The lower insertion efficiency was expected, as the *ben-1b* sgRNA appears to be less efficient than *ben-1a* in generating double-strand DNA break by CRISPR–Cas9 (Figure 2E). We confirmed the gene conversion event by amplifying the *ben-1* locus by PCR and sequencing it.

## DISCUSSION

CRISPR provides a versatile way to engineer the *C. elegans* genome. Its power stems from the ability to target the Cas9 nuclease to any genomic location by simply constructing the appropriate short guide RNA. In principle, the approach is limited only by the requirement of a PAM, NGG, although further work is required to establish how targeting efficiency varies with genome location. The CRISPR approach offers advantages compared with MosTIC, the pioneering state-of-the-art technology for transgene-mediated gene conversion in *C. elegans* (24). Most significantly, MosTIC requires a pre-existing strain harboring a *Mos* transposon insertion ∼1 kb away from the site to be engineered. Extensive efforts have been made to create libraries of strains with *Mos*1 insertions (25). However, finding a strain with an appropriately located *Mos*1 insertion can still be problematic, particularly since the efficiency with which changes located on transgenes are copied by gene conversion falls rapidly with distance from the double-strand break induced by transposon excision (24).

The CRISPR-mediated gene conversion we used as proof of principle involves insertion of an HygR gene cassette as a positive selection marker. In principle, the method can be extended to any transgene sequence, allowing introduction of fluorescent tags or specific base changes that can be identified by PCR, or PCR followed by restriction analyses. We envisage that incorporation of an HygR cassette within the homologous sequence arms could be used to facilitate identification both of CRISPR–Cas9-induced mutations that have no known phenotype, and when making specific sequence changes or inserting a GFP or antibody tag. In cases where the HygR cassette could compromise the function of the modified gene, the cassette could be flanked with FRT or LoxP sites, to enable its subsequent removal following germline expression of the appropriate recombinase. By careful design it should be possible to leave only an FRT/LoxP site that is localized in an intron as a legacy of the hygromycin cassette. Also note that it may be useful to disrupt the PAM sequence in the repair construct, to prevent further double-strand breaks following gene conversion, although we did not do this in our experiments. Finally, although in our experiments we cloned the repair template on a plasmid, it should be possible to use PCR products for the same purpose.

Use of hygromycin selection has only been introduced recently in *C. elegans* (28), but we find it provides very efficient selection at low cost. Importantly, resistance is conferred as a dominant trait, selection is fast (4 days) and efficient, and does not require a specific genetic background. Incorporating the PEEL-1 toxin pioneered previously for MosTIC (32) into our scheme and using multiple mCherry co-injection markers facilitate selection against false positives in which gene conversion has not occurred.

The efficiency in generating mutations by CRISPR–Cas9 system varies with targeting sequence (Figure 2E), something that has been observed previously (11). The reasons for this difference are unclear, but most likely are due to the protospacer sequences targeted by the CRISPR–Cas9 system. At some loci, low efficiency in generating double-strand DNA breaks may limit the usefulness of this technology to isolate gene conversion events. As more data are collected, it may be possible to use predictive algorithms to design sgRNAs that efficiently target DNA cleavage.

In summary, we have shown that the CRISPR–Cas9 system can be used in *C. elegans* as an efficient tool both to generate targeted mutations and to insert desired sequences by homologous recombination at a specific site, with comparative ease. The only limitations are the requirement for an NGG PAM sequence at the edited site and the potential of off-target effects, both of which have been addressed elsewhere [7, 8, 11]. We anticipate that CRISPR–Cas9-targeted knock-ins will become an important tool to dissect gene and genome function in *C. elegans*.

## SUPPLEMENTARY DATA

Supplementary Data are available at NAR Online.

## FUNDING

European Molecular Biology Organization Fellowship [ALTF 1098-2011 to C.C.]; Medical Research Council Core Funding; European Research Council Advanced Grant [Proposal No 269058–ACMO]. Funding for open access charge: European Research Council Advanced Grant.

*Conflict of interest statement*. None declared.

## Supplementary Material

Supplementary Data
