# Supplementary material for: Efficient genome editing in Caenorhabditis elegans by CRISPR-targeted homologous recombination
Source: Nucleic Acids Res. 2013 Sep 5;41(20):e193. doi: 10.1093/nar/gkt805 (PMC3814388; doi:10.1093/nar/gkt805)
Supplement: Supplementary Data [file supp_41_20_e193__index.html]

Efficient genome editing in Caenorhabditis elegans by CRISPR-targeted homologous recombination — Efficient genome editing in Caenorhabditis elegans by CRISPR-targeted homologous recombination — Supplementary Data 

# Efficient genome editing in *Caenorhabditis elegans* by CRISPR-targeted homologous recombination

## Supplementary Data

files

**Files in this Data Supplement:**

- Supplementary Data - pdf file
