# Supplementary material for: Efficient genome editing in Caenorhabditis elegans by CRISPR-targeted homologous recombination
Source: Nucleic Acids Res. 2013 Sep 5;41(20):e193. doi: 10.1093/nar/gkt805 (PMC3814388; doi:10.1093/nar/gkt805)
Supplement: Supplementary Data [file supp_gkt805_nar-01937-met-h-2013-File005.pdf]

Codon-optimized Cas9 sequence

ATGGACTACAAAGATCACGATGGCGACTACAAAGATCACGATATCGACTACAAAGACGATGACGATAAACCAAA
GAAGAAGCGTAAGGTAGACAAGAAGTACTCCATCGGACTCGACATCGGAACCAACTCCGTCGGATGGGCCGTCAT
CACCGACGACTACAAGGTCCCATCCAAGAAGTTCAAGGTCCTCGGAAACACCGACCGTCACTCCATCAAGAAGAA
CCTCATCGGAGCCCTCCTCTTCGGATCCGGAGAGACCGCCGAGGCCACCCGTCTCAAGCGTACCGCCCGTCGTCGT
TACACCCGTCGTAAGAACCGTATCTGCTACCTCCAAGAGATCTTCTCCAACGAGATGGCCAAGGTCGACGACTCCT
TCTTCCACCGTCTCGAGGAGTCCTTCCTCGTCGAGGAGGACAAGAAGCACGAGCGTCACCCAATCTTCGGAAACA
TCGTCGACGAGGTCGCCTACCACGAGAAGTACCCAACCATCTACCACCTCCGTAAGAAGCTCGCCGACTCCACCG
ACAAGGCCGACCTCCGTCTCATCTACCTCGCCCTCGCCCACATGATCAAGTTCCGTGGACACTTCCTCATCGAGGG
AGACCTCAACCCAGACAACTCCGACGTCGACAAGCTCTTCATCCAACTCGTCCAAATCTACAACCAACTCTTCGAG
GAGAACCCAATCAACGCCTCCCGTGTCGACGCCAAGGCCATCCTCTCCGCCCGTCTCTCCAAGTCCCGTCGTCTCG
AGAACCTCATCGCCCAACTCCCAGGAGAGAAGCGTAACGGACTCTTCGGAAACCTCATCGCCCTCTCCCTCGGACT
CACCCCAAACTTCAAGTCCAACTTCGACCTCGCCGAGGACGCCAAGCTCCAACTCTCCAAGGACACCTACGACGA
CGACCTCGACAACCTCCTCGCCCAAATCGGAGACCAATACGCCGACCTCTTCCTCGCCGCCAAGAACCTCTCCGAC
GCCATCCTCCTCTCCGACATCCTCCGTGTCAACTCCGAGATCACCAAGGCCCCACTCTCCGCCTCCATGATCAAGC
GTTACGACGAGCACCACCAAGACCTCACCCTCCTCAAGGCCCTCGTCCGTCAACAACTCCCAGAGAAGTACAAGgt
aagtttaaacatatatatactaactaaccctgattatttaaattttcagGAGATCTTCTTCGACCAATCCAAGAACGGATACGCCGGATACATCGAC
GGAGGAGCCTCCCAAGAGGAGTTCTACAAGTTCATCAAGCCAATCCTCGAGAAGATGGACGGAACCGAGGAGCT
CCTCGTCAAGCTCAACCGTGAGGACCTCCTCCGTAAGCAACGTACCTTCGACAACGGATCCATCCCACACCAAATC
CACCTCGGAGAGCTCCACGCCATCCTCCGTCGTCAAGAGGACTTCTACCCATTCCTCAAGGACAACCGTGAGAAG
ATCGAGAAGATCCTCACCTTCCGTATCCCATACTACGTCGGACCACTCGCCCGTGGAAACTCCCGTTTCGCCTGGA
TGACCCGTAAGTCCGAGGAGACCATCACCCCATGGAACTTCGAGGAGGTCGTCGACAAGGGAGCCTCCGCCCAAT
CCTTCATCGAGCGTATGACCAACTTCGACAAGAACCTCCCAAACGAGAAGGTCCTCCCAAAGCACTCCCTCCTCTA
CGAGTACTTCACCGTCTACAACGAGCTCACCAAGGTCAAGTACGTCACCGAGGGAATGCGTAAGCCAGCCTTCCT
CTCCGGAGAGCAAAAGAAGGCCATCGTCGACCTCCTCTTCAAGACCAACCGTAAGGTCACCGTCAAGCAACTCAA
GGAGGACTACTTCAAGAAGATCGAGTGCTTCGACTCCGTCGAGATCTCCGGAGTCGAGGACCGTTTCAACGCCTC
CCTCGGAGCCTACCACGACCTCCTCAAGATCATCAAGGACAAGGACTTCCTCGACAACGAGGAGAACGAGGACAT
CCTCGAGGACATCGTCCTCACCCTCACCCTCTTCGAGGACCGTGGAATGATCGAGGAGCGTCTCAAGACCTACGCC
CACCTCTTCGACGACAAGGTCATGAAGCAACTCAAGCGTCGTCGTTACACCGGATGGGGACGTCTCTCCCGTAAG
CTCATTAACGGAATCCGTGACAAGCAATCCGGAAAGgtaagtttaaacagttcggtactaactaaccatacatatttaaattttcagACCATCCTCG
ACTTCCTCAAGTCCGACGGATTCGCCAACCGTAACTTCATGCAACTCATCCACGACGACTCCCTCACCTTCAAGGA
GGACATCCAAAAGGCCCAAGTCTCCGGACAAGGACACTCCCTCCACGAGCAAATCGCCAACCTCGCCGGATCCCC
AGCCATCAAGAAGGGAATCCTCCAAACCGTCAAGATCGTCGACGAGCTCGTCAAGGTCATGGGACACAAGCCAG
AGAACATCGTCATCGAGATGGCCCGTGAGAACCAAACCACCCAAAAGGGACAAAAGAACTCCCGTGAGCGTATG
AAGCGTATCGAGGAGGGAATCAAGGAGCTCGGATCCCAAATCCTCAAGGAGCACCCAGTCGAGAACACCCAACT
CCAAAACGAGAAGCTCTACCTCTACTACCTCCAAAACGGACGTGACATGTACGTCGACCAAGAGCTCGACATCAA
CCGTCTCTCCGACTACGACGTCGACCACATCGTCCCACAATCCTTCATCAAGGACGACTCCATCGACAACAAGGTC
CTCACCCGTTCCGACAAGAACCGTGGAAAGTCCGACAACGTCCCATCCGAGGAGGTCGTCAAGAAGATGAAGAAC
TACTGGCGTCAACTCCTCAACGCCAAGCTCATCACCCAACGTAAGTTCGACAACCTCACCAAGGCCGAGCGTGGA
GGACTCTCCGAGCTCGACAAGGCCGGATTCATCAAGCGTCAACTCGTCGAGACCCGTCAAATCACCAAGCACGTC
GCCCAAATCCTCGACTCCCGTATGAACACCAAGTACGACGAGAACGACAAGCTCATCCGTGAGGTCAAGGTCATC
ACCCTCAAGTCCAAGCTCGTCTCCGACTTCCGTAAGGACTTCCAATTCTACAAGGTCCGTGAGATCAACAACTACC
ACCACGCCCACGACGCCTACCTCAACGCCGTCGTCGGAACCGCCCTCATCAAGAAGTACCCAAAGCTCGAGTCCG
AGTTCGTCTACGGAGACTACAAGGTCTACGACGTCCGTAAGATGATCGCCAAGTCCGAGCAAGAGATCGGAAAGgt
aagtttaaacatgattttactaactaactaatctgatttaaattttcagGCCACCGCCAAGTACTTCTTCTACTCCAACATCATGAACTTCTTCAAGA
CCGAGATCACCCTCGCCAACGGAGAGATCCGTAAGCGTCCACTCATCGAGACCAACGGAGAGACCGGAGAGATC
GTCTGGGACAAGGGACGTGACTTCGCCACCGTCCGTAAGGTCCTCTCCATGCCACAAGTCAACATCGTCAAGAAG
ACCGAGGTCCAAACCGGAGGATTCTCCAAGGAGTCCATCCTCCCAAAGCGTAACTCCGACAAGCTCATCGCCCGT
AAGAAGGACTGGGACCCAAAGAAGTACGGAGGATTCGACTCCCCAACCGTCGCCTACTCCGTCCTCGTCGTCGCC
AAGGTCGAGAAGGGAAAGTCCAAGAAGCTCAAGTCCGTCAAGGAGCTCCTCGGAATCACCATCATGGAGCGTTCC
TCCTTCGAGAAGAACCCAATCGACTTCCTCGAGGCCAAGGGATACAAGGAGGTCAAGAAGGACCTCATCATCAAG
CTCCCAAAGTACTCCCTCTTCGAGCTCGAGAACGGACGTAAGCGTATGCTCGCCTCCGCCGGAGAGCTCCAAAAG
GGAAACGAGCTCGCCCTCCCATCCAAGTACGTCAACTTCCTCTACCTCGCCTCCCACTACGAGAAGCTCAAGGGAT
CCCCAGAGGACAACGAGCAAAAGCAACTCTTCGTCGAGCAACACAAGCACTACCTCGACGAGATCATCGAGCAA
ATCTCCGAGTTCTCCAAGCGTGTCATCCTCGCCGACGCCAACCTCGACAAGGTCCTCTCCGCCTACAACAAGCACC
GTGACAAGCCAATCCGTGAGCAAGCCGAGAACATCATCCACCTCTTCACCCTCACCAACCTCGGAGCCCCAGCCG
CCTTCAAGTACTTCGACACCACCATCGACCGTAAGCGTTACACCTCCACCAAGGAGGTCCTCGACGCCACCCTCAT
CCACCAATCCATCACCGGACTCTACGAGACCCGTATCGACCTCTCCCAACTCGGAGGAGACATGAGCCGTAGACG
AAAAGCGAATCCGACAAAACTGAGTGAAAACGCGAAGAAGCTTGCCAAGGAAGTTGAAAATTAA

: 3X FLAG

: SV40

: Artificial intron

: *egl-13*

Chimeric RNA sequence with *rpr-1* promoter

cgatttcggctaaaaaatagcgaaaaaacatcaaatttgtattaaaaagaagcagttgaaattttgagtgaggctcagagact
acaaactacaaaaaggctcagcctcaaccaatttttagtgttaaaaatttgattttttcagttaaaaacgatggtttttgatgcttttt
acctattttacagatagaaaattttaaatttcgcaaaaatctttaaaaataaactttttttgtttggttttcgctcgaaaatagctttaaa
tttgcagtttttctctcaaaaactctaaatttcatgcgttccacgtggctttctatcaatttaaatactaatattaatattttcttcaaaa
tctgccacgttacagcctgccatagccttctgaaactctacaattcccctctaatcactagcgccgcctccccgcacactcaa
cacagcgagcggcggaacccgcggggtgtcggccgctcggacacgctcccgtatataacgacgcggcgcgcgtcaag
ttgtGNNNNNNNNNNNNNNNNNNNGTTTTAGAGCTAGAAATAGCAAGTTAA
AATAAGGCTAGTCCGTTATCAACTTGAAAAAGTGGCACCGAGTCGGTGCTt
ttgtgggcttcaggctttctgttgggccgttttaggccaaatgatagaaagccacgtggaaatcatgaatttaaagctattttcg
agcgaaaatcagaaaaaaagtttatttttaaagatttttgctgaattttacattttctaaatgtaaaataggtgaaaagcatcaaa
aaccatagtttttaactgaaaaaatcaaatttttaacactaaaaattggttgaggctgatcctttttgtagtttgtagtctctgagcc
tcactcaaaatttcaaaagttctttttgatacaaatttgatgatttttcgctatttttctagccgaaatcgtatttttcaacattttcttaa
caaaattaggatttgctcaaaattgatataacattatttaaaagtaccgttaaagttgtaccaaaaatagtggaaatttatttttttt

: *rpr-1* promoter

: target RNA sequence, which is replaced by EcoRI

: guide RNA scaffold sequence

: *rpr-1* terminator

Chimeric RNA sequence with U6 promoter

Cccaaatgtttttataatttggcaaattaccaaaaatgtgactggaagcattaacaaaattttgaacaatattcgaggtttcaaa
atcatgatattttagatattttggactgttttgagtcaaattgttaatgacagcattttaagagctcccaacacatagtgtttccaat
gttatacccaatcaataatagcaagtcaataaactacctctacactatttctggtaataggcgaaacctctacactgtcagtcac
tttgtaaggtgtgcctatgtatttccataatatttcatacaaattGNNNNNNNNNNNNNNNNNNNGTTT
TAGAGCTAGAAATAGCAAGTTAAAATAAGGCTAGTCCGTTATCAACTTGA
AAAAGTGGCACCGAGTCGGTGCTttttttgactaatttctggtattattgtttagaattgtaagaaaatttta
cattccagaaaatgtatccaaaaaacttacgcaaactcggagcgtatagttgcatgaagtgctggatccgaaatacgaacga
caaattgttgatactttgctttcaaactctcgtaataaggcatcacaaagttctcaatttt

: U6 promoter

: target RNA sequence, which is replaced by HindIII

: guide RNA scaffold sequence

: U6 terminator
